# Mapping PAM4 (clivatuzumab), a monoclonal antibody in clinical trials for early detection and therapy of pancreatic ductal adenocarcinoma, to MUC5AC mucin

**DOI:** 10.1186/1476-4598-12-143

**Published:** 2013-11-20

**Authors:** David V Gold, Guy Newsome, Donglin Liu, David M Goldenberg

**Affiliations:** 1 Garden State Cancer Center, Center for Molecular Medicine and Immunology, Morris Plains, NJ, USA; 2 Immunomedics, Inc., Morris Plains, NJ, USA

**Keywords:** Pancreatic cancer, Early detection, PAM4, MUC5AC, Clivatuzumab, Enzyme immunoassay

## Abstract

**Background:**

PAM4, an antibody that has high specificity for pancreatic ductal adenocarcinoma (PDAC), compared to normal pancreas, benign lesions of the pancreas, and cancers originating from other tissues, is being investigated as a biomarker for early detection, as well as antibody-targeted imaging and therapy. Therefore, the identity of the antigen bound by this monoclonal antibody (MAb) can provide information leading to improved use of the antibody. Prior results suggested the antigen is a mucin-type glycoprotein rich in cysteine disulfide bridges that provide stable conformation for the PAM4-epitope.

**Methods:**

Indirect and sandwich enzyme immunoassays (EIA) were performed to compare and contrast the reactivity of PAM4 with several anti-mucin antibodies having known reactivity to specific mucin species (e.g., MUC1, MUC4, MUC5AC, etc.). Studies designed to block reactivity of PAM4 with its specific antigen also were performed.

**Results:**

We demonstrate that MAbs 2-11 M1 and 45 M1, each reactive with MUC5AC, are able to provide signal in a heterologous sandwich immunoassay where PAM4 is the capture antibody. Further, we identify MAbs 21 M1, 62 M1, and 463 M1, each reactive with MUC5AC, as inhibiting the reaction of PAM4 with its specific epitope. MAbs directed to MUC1, MUC3, MUC4, MUC16 and CEACAM6 are not reactive with PAM4-captured antigen, nor are they able to block the reaction of PAM4 with its antigen.

**Conclusions:**

These data implicate MUC5AC as a specific mucin species to which PAM4 is reactive. Furthermore, this realization may allow for the improvement of the current PAM4 serum-based immunoassay for detection of early-stage PDAC by the application of anti-MUC5AC MAbs as probes in this sandwich EIA.

## Background

Mucin glycoproteins are high molecular weight, heavily glycosylated, proteins that include at least 19 species categorized on the basis of their unique protein cores, and can be found as either transmembrane components of the cell or as secreted products. Abnormal expression of mucins is a well-known occurrence in many forms of cancer (see reviews [1]-3]), including pancreatic ductal adenocarcinoma (PDAC) [4-6]. Neo-expression and/or upregulation/downregulation of specific mucin species, with and without the generation of newly transcribed and translated splice variants [7], have been well-documented in the literature. Alteration of carbohydrate moieties through the addition of new terminal sugars (e.g., neuraminic acids), underglycosylation, and other abnormal biochemical pathways also have been observed [8-10]. These modifications may lead to changes in conformational structure and/or appearance or disappearance of specific epitopes. Additionally, changes may be observed for the intracellular distribution of the mucin species under consideration, such as MUC1, which in normal tissues is a transmembrane glycoprotein, but with neoplastic transformation is found in the cytoplasm as well [11,12]. These events may prove to be of biological and clinical significance in the process of neoplastic development and progression, as well as provide new biomarkers/targets for early detection and targeted therapy of cancer.

Our laboratory initially reported the use of a polyclonal antiserum to identify a pancreatic ductal mucin, which at the level of sensitivity provided by indirect immunohistochemistry (IHC), was shown to contain an epitope relatively specific to the pancreas [13], and ultimately resulted in the development of monoclonal antibody (MAb), PAM4 [14], also known as clivatuzumab, the humanized form. PAM4 demonstrates high specificity for PDAC with little to no reactivity towards normal and benign, non-neoplastic, pancreatic tissues, although it does show limited reactivity (approximately 10% of all specimens examined) with adenocarcinomas originating in certain other organs (e.g., stomach, colon, lung) [14-16]. PAM4 identifies a biomarker that, if present, provides a high diagnostic likelihood of the presence of pancreatic neoplasia [16-18]. Thus, clinical applications for detection of early-stage disease [16,18], and antibody-targeted imaging and therapy, are being pursued [19,20]. In addition to PDAC, the PAM4-biomarker is expressed in the precursor lesions, pancreatic intraepithelial neoplasia (PanIN, including the earliest developing lesion, PanIN-1A), and intraductal papillary mucinous neoplasia (IPMN), suggesting that there may be oncogenic significance to its expression [15]. In the current study, we investigated the identity of the mucin species to which this clinically-relevant antibody is reactive, in order to understand what role this mucin may play in the development and progression of pancreatic cancers.

## Results

**Figure 1:**
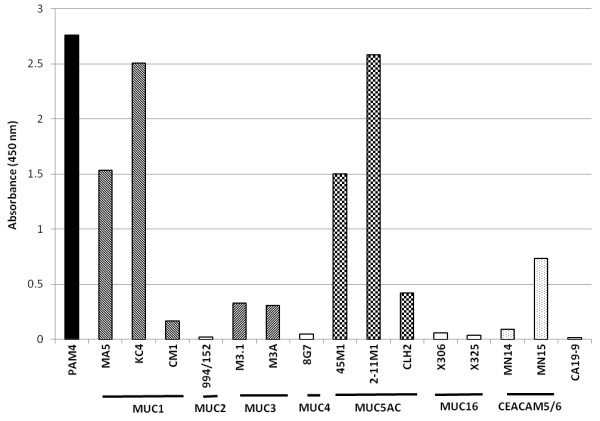
**Reactivity of several anti-mucin MAbs with a high molecular weight mucin containing fraction (CPM1) isolated from the Capan-1 human pancreatic adenocarcinoma.** MAbs are identified by clone name with reactive species of mucin indicated by horizontal bars beneath MAb clone names (MUC1, etc.). In addition to PAM4, substantial reactions were observed for anti-MUC1, -MUC5AC, and -CEACAM6 antibodies. All MAbs were employed at a concentration of 10 μg/mL.

Several MAbs were evaluated by the indirect EIA for reactivity with plates coated with CPM1 (Figure 1), a high molecular weight mucin fraction isolated from the Capan-1 human pancreatic cancer xenograft. Murine PAM4 and MAbs reactive specifically with MUC1 and MUC5AC mucins provided elevated reactivity in this indirect immunoassay, with minor reactivity also observed for MAbs directed to MUC3 and CEACAM6. Essentially no reaction was seen with MAbs to MUC2, MUC4, MUC16, and CEACAM5 glycoproteins, or the CA19-9 carbohydrate epitope. It should be noted that a negative EIA reaction does not necessarily indicate absence of the mucin-antigen, because the specific epitope structure may be present, but inaccessible (i.e., cryptic). This is likely the case for MAb-CLH2 anti-MUC5AC generated against a peptide derived from the mucin's tandem repeat [21], since the other two anti-MUC5AC MAbs are highly reactive. Similarly, CM1 anti-MUC1 was considerably less reactive than MA5 and KC4 anti-MUC1 antibodies. Capan-1 cells produce well-differentiated tumors with highly glycosylated mucins. Thus, it is likely that both CLH2 and CM1, reactive with the tandem repeat domains of their respective mucins, would not be reactive with CPM1, since the tandem repeat epitopes are inaccessible.

**Figure 2:**
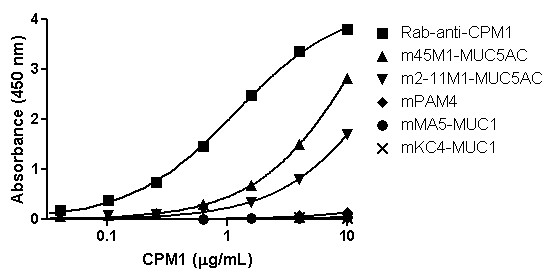
**Reaction of several anti-mucin MAbs with PAM4-captured antigen.** Mucin antigens were captured on hPAM4 coated plates, and then probed with several murine anti-mucin MAbs for reaction signal. Both anti-MUC5AC MAbs (2-11 M1 and 45 M1) bound to the hPAM4-captured mucin, whereas the anti-MUC1 MAbs (MA5 and KC4) did not bind. The homologous hPAM4/mPAM4, capture/probe immunoassay gave no signal, suggesting the density of PAM4 epitopes within the mucin may be low, possibly only a single site. A rabbit polyclonal anti-CPM1 IgG was used as a positive control for reaction with hPAM4-captured antigen.

We then evaluated whether the anti-mucin MAbs were reactive with PAM4-captured mucin. Humanized PAM4 (hPAM4; clivatuzumab)-coated plates were used to capture the specific mucin-antigen from the CPM1 fraction, which was then probed with various anti-mucin MAbs. Murine MAbs (mMAbs) specifically reactive with MUC1, MUC3, MUC4, MUC16 and CEACAM6 did not provide a signal in these heterologous sandwich immunoassays. On the other hand, both anti-MUC5AC mMAbs tested, 45 M1 and 2-11 M1, gave positive reactions with the hPAM4-captured antigen (Figure 2), with 45 M1 showing significantly greater reaction than 2-11 M1 (Kd = 14.32 ± 1.08 μg/mL and 24.4 ± 7.83 μg/mL, respectively, for MAbs 45 M1 and 2-11 M1; *P* < 0.001). However, neither of these individual anti-MUC5AC MAbs provided as strong signal intensity as the rabbit anti-CPM1 polyclonal IgG fraction. Importantly, mPAM4 did not bind to the hPAM4-captured antigen, nor did hPAM4 bind to mPAM4-captured antigen, suggesting that the PAM4 epitope is present at low density, possibly only a single site within the mucin-antigen.

**Figure 3:**
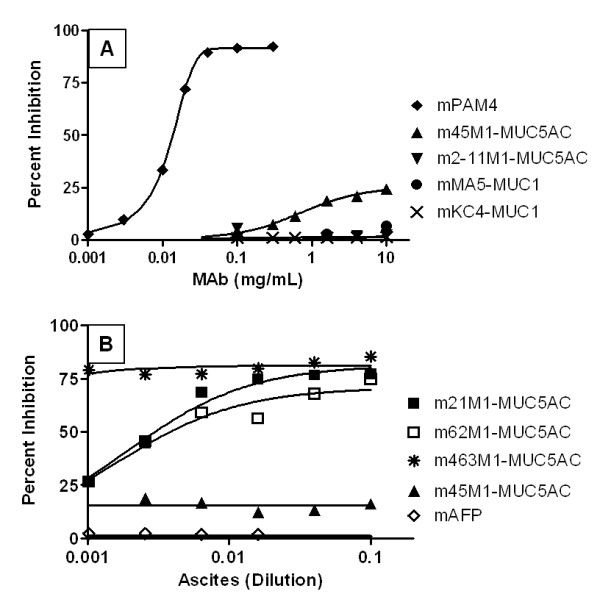
**Inhibition of hPAM4/antigen binding reaction by murine anti-mucin MAbs. A)** Anti-mucin mMAbs (purified IgG) were added to CPM1-coated plates as potential inhibitors prior to addition of hPAM4. mPAM4 provided almost complete inhibition of the reaction between hPAM4 and antigen with the 45 M1 anti-MUC5AC providing limited inhibitory effect (IC_max_ = 25.5%). Neither 2-11 M1, anti-MUC5AC nor MA5 and KC4, anti-MUC1 MAbs were able to inhibit the specific hPAM4/antigen reaction. **B)** A similar inhibition study was performed with several anti-MUC5AC MAbs obtained as ascites fluids. MAbs 21 M1, 62 M1, and 463 M1, anti-MUC5AC provided substantial inhibitory effect, similar to that observed with mPAM4 IgG self-inhibition. The ascites form of 45 M1 yielded an inhibitory effect similar to that of the purified IgG. Ascites containing anti-alpha fetoprotein was employed as a negative control.

Follow-up studies were designed to inhibit the binding of hPAM4 to CPM1-coated plates (Figure 3). Although 2-11 M1 anti-MUC5AC was unable to inhibit hPAM4-CPM1 binding, 45 M1 anti-MUC5AC did provide a limited inhibitory effect, with IC_max_ = 25.5% inhibition. mPAM4, included as a positive control, provided IC_max_ = 92.4% self-inhibition at a concentration 0.1 μg/mL, while the MA5 and KC4 anti-MUC1 antibodies provided no inhibition, even at the highest concentration evaluated (10 μg/mL). hPAM4 was unable to completely block mPAM4 binding to the CPM1 antigen (IC_max_ = 52.8%), a not unexpected finding since the humanized version of PAM4 may have a lower affinity than the murine parent. Ascites fluids containing mMAbs with known mapping to MUC5AC were serially diluted as inhibitory reagents, with results shown in Figure 3B. mMAbs 21 M1, 62 M1, and 463 M1 each provided inhibition similar to the results shown for mPAM4 self-blocking, with 45 M1 ascites providing limited inhibition, similar to what was observed with the commercially available 45 M1-IgG. Ascites fluid containing a murine anti-alpha-fetoprotein (AFP) MAb, included here as a negative control, provided no inhibition of the hPAM4 binding to CPM1. Unfortunately, insufficient volumes of ascites precluded determination of MAb concentrations, so that relative blocking efficiency could not be calculated.

Epitopes for MAbs 21 M1, 62 M1, and 463 M1 have each been mapped to the C-terminal region of MUC5AC (22). This suggested that PAM4 may also be reactive with the C-terminal region of the mucin. Thus, preliminary studies were conducted to transfect the human CFPAC pancreatic cancer cell line with a plasmid encoding the peptide M-MUC5AC-CH-long (23), from the C-terminal region of MUC5AC. Western blots of the expressed peptide showed positive reactivity for the control myc-tag and the 45 M1 epitope, but not PAM4 (data not shown).

## Discussion

**Figure 4:**
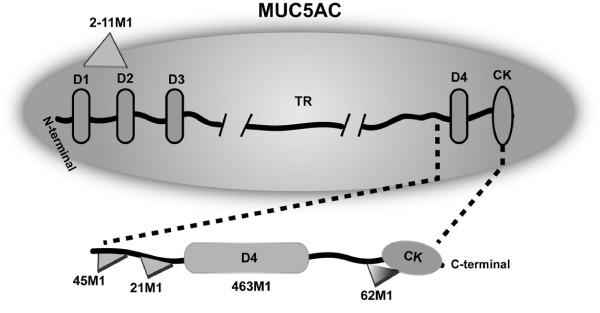
**Representation of the domains of the MUC5AC glycoprotein with reactive epitopes indicated for several anti-MUC5AC MAbs.** (Derived from references [22,23,28]). Data derived by transfection with plasmid vectors containing the cDNA of the 3’-end of MUC5AC, along with derivative cDNA vectors obtained by restriction enzyme digestion, have identified the location of specific epitopes for anti-MUC5AC MAbs employed in the current studies. Specific blocking studies (Figures 3A and B) suggest the PAM4-epitope resides within the cysteine-rich C-terminus domain.

The current studies suggest that PAM4 is reactive with the MUC5AC mucin glycoprotein. Figure 4 presents a map of the MUC5AC mucin domains with reactive epitopes indicated for several of the anti-MUC5AC MAbs employed in our studies [22-24]. CLH2 is reactive with the peptide core of the tandem repeat domain [21], and is likely a cryptic epitope within the Capan-1 tumor-derived MUC5AC. 2-11 M1 is reactive with the N-terminus of the mucin [23], and 45 M1 at the furthest N-terminal region of the cysteine-rich, C-terminus [24]. Both of these MAbs were reactive with PAM4-captured mucin, whereas MAbs to MUCs 1, 3, 4, and 16 were not. We observed that 45 M1 provides a significantly greater signal response than 2-11 M1, suggesting a greater density of 45 M1-epitopes than 2-11 M1-epitopes within CPM1. However, this may simply be due to a loss of 2-11 M1 epitopes through proteolytic digestion of the relatively non-glycosylated N-terminus, and/or molecular shear of this very large glycoprotein during purification. In any case, the 2-11 M1 antibody provided no inhibition of the hPAM4-CPM1 interaction, suggesting the epitope is located distant to the PAM4-epitope. On the other hand, 45 M1 did inhibit the hPAM4-CPM1 interaction, albeit only partially, suggesting that the PAM4-epitope is within the C-terminal region of the mucin or conformationally altered by interaction of this antibody with the mucin molecule. MAbs 21 M1, 62 M1, and 463 M1 also have been mapped to the C-terminal region of the MUC5AC mucin [22-24], and each provided significant inhibition of the PAM4-mucin reaction. Taken together, our data provide direct evidence that PAM4 is reactive with the identical mucin (MUC5AC), and that the PAM4 epitope is either directly-blocked, or conformationally modified, by interaction of these MAbs with the MUC5AC antigen.

Preliminary studies to confirm the C-terminus location of the PAM4 epitope by transfection with a plasmid encoding the C-terminal peptide were, as yet, unrevealing. Whereas the control anti-myc tag and 45 M1 antibodies showed positive reactions with the expressed peptide, PAM4 was negative. These data would suggest the PAM4-epitope is different from the 45 M1-epitope, and not located within the C-terminal region. However, as with the EIAs described in the manuscript, a positive signal can provide a meaningful interpretation, the specific epitope is expressed, but a negative response does not necessarily provide useful information. There are several possible explanations as to why PAM4 might give a negative response: 1) The epitope conformation may be altered in the expressed peptide as compared to the native mucin, 2) the epitope may include carbohydrate that may not be present and/or may be altered from the native state, and 3) the epitope may reside within a non-C-terminus domain, but is conformationally altered by reaction of MAbs with the C-terminus, amongst several other possibilities. We intend to continue exploration of these factors in an effort to better define the PAM4-epitope.

We had initially reported that PAM4 was reactive with the MUC1 mucin species [15,17,25]. This was based upon *MUC1*-gene transfection studies, where PAM4 was observed to react with the gene-transfected MUC1^+^ cell line, but not the MUC1^-^ parental cell line or vector control cell lines. However, other evidence acquired since then has questioned this interpretation, suggesting that MUC1 transfection may have upregulated other mucins as well. Prior results from our laboratory lend support to the current findings. The PAM4 epitope was found to be highly sensitive to mild reduction with dithiothreitol (0.02 M, 15 min, 20°C) or heat (100°C, 2 min), suggesting the epitope is peptide in nature, and highly dependent upon a specific conformation of the protein core kept intact by disulfide bridges [14]. This is unlikely to be MUC1 with all of the cysteines located within the transmembrane domain of the mucin, but is consistent with the loss of reactivity shown by several anti-MUC5AC MAbs upon reduction of the mucin antigen. Further, employing immunohistochemical methods, we reported that frequency of expression and morphologic distribution of the PAM4-epitope within PDAC and its precursor lesions shared greater similarity to those described for MUC5AC than for MUC1 [15].

As already noted, there is an extensive literature regarding the abnormal expression of mucin-type glycoproteins in association with, or as a consequence of, the development and progression of malignancy [1-12]. Aspects of this research effort have been directed towards both an understanding of the potential role of mucins in tumor biology, and as a means to identify what is hoped to be clinically-relevant biomarkers/targets for early detection and therapy of malignancy. Unfortunately, there exists a considerable amount of contradictory information regarding expression of specific mucin species, due mostly to differences in the methods used to detect and identify the mucin. Detection of mucins by MAbs reactive to different epitopes within the identical mucin species or use of different primer sets for RT-PCR can provide contradictory results for expression of mucin species within malignant and benign lesions. For the present study, this is highlighted by the positive response of MAbs PAM4, 2-11 M1, and 45 M1 with CPM1-derived MUC5AC, in contrast to the limited response obtained with MAb-CLH2. Considering that Capan-1 is a well-differentiated tumor with highly glycosylated mucins [26], it is not unexpected that the underlying tandem repeat peptide identified by CLH2 might be cryptic and therefore not detectable. Thus, expression of specific mucin species is related to the method used for its detection.

Both the 2-11 M1 and 45 M1 MAbs were generated against a mucin derived from a human ovarian mucinous cyst [23,27], CLH2 [21] was generated against the MUC5AC tandem repeat peptide, and each is able to discriminate normal and malignant pancreatic tissues by immunohistochemistry. High frequency detection of PDAC and limited to no reactivity with normal pancreas tissue has provided considerable interest for use of MUC5AC as a biomarker of PDAC. However, MUC5AC, as detected by these MAbs, does not show organ specificity, and is expressed within several normal adult tissues (e.g., gastric, colonic and lung mucosa, amongst others), and to varying extent within malignant lesions derived from these tissues and others (e.g., lung adenocarcinoma and PDAC).

Although not directly compared in a single assay format with the same specimen set, it appears that the PAM4-epitope demonstrates a higher specificity to discriminate malignant from benign, non-neoplastic lesions of the pancreas and, importantly, cancers originating from other organs, than these other MAbs. The specific structural element of the PAM4-epitope responsible for this is of particular interest. We have presented considerable evidence, including immunohistochemical studies of tissue specimens [14-16], as well as results from immunoassays of patient sera [16-18], showing that PAM4 is reactive with a biomarker that may have clinical relevance for the detection and diagnosis of early-stage PDAC. The current serum-based immunoassay employs PAM4 as the capture component and a polyclonal anti-CPM1 IgG as the probe. With the information provided from the present studies, we may be able to substitute anti-MUC5AC MAbs (in particular, the 45 M1 MAb, since the PAM4 and 45 M1 epitopes are clearly distinct, yet both present within the same molecule), for the rabbit polyclonal anti-CPM1 IgG currently employed as the probe. This could provide a more suitable immunoassay for clinical use, since the rabbit polyclonal is limited in quantity with potential problems in lot-to-lot consistency, whereas the anti-MUC5AC MAbs are well defined and available for consistent application. However, the anti-MUC5AC MAbs would have to at least match the sensitivity and specificity provided by the polyclonal probe. Such studies are in progress.

## Conclusions

The use of MAbs having defined reactivity with MUC5AC has identified two that are able to provide a signal response in the heterologous PAM4 sandwich EIA, and three that are able to inhibit the interaction between PAM4 and its mucin antigen. These data implicate MUC5AC as the antigen to which PAM4 is reactive. However, it should be pointed out that these studies do not negate the possibility that PAM4 is also reactive with other MUC-species. It is certainly possible that two or more MUC-species can share the same epitope. The significance of our current finding is that MUC5AC contains an epitope structure which is newly expressed early in the development of pancreatic neoplasia. Whether or not this epitope structure represents a reactive site for activation of oncogenesis in the pancreas is as yet unknown. Nevertheless, this epitope can serve as a biomarker for PDAC, as well as a target for antibody-directed imaging and therapy.

## Methods

### Antigen and antibodies

A mucin-containing fraction, designated CPM1, was isolated, as described previously [17], from the Capan-1 human PDAC xenograft in athymic nude mice. Briefly, this consisted of homogenization of the dissected tumor in 0.1 M ammonium bicarbonate containing 0.5 M sodium chloride. Following high-speed centrifugation (20,000 *g* × 45 min), the soluble material was chromatographed on a Sepharose 4B-CL column, and then eluted with the identical ammonium bicarbonate-sodium chloride solution. The void volume material was collected, dialyzed against 0.01 M sodium phosphate, pH 7.2, and then passed through hydroxyappatite to remove nucleic acids and proteins. The non-binding, mucin-containing fraction was again dialyzed extensively to remove salts and used as a source of antigen.

Antibodies used in the current study are listed in Table 1 with clone and source information. For sandwich and blocking studies, PAM4 was available in both murine (mPAM4) and humanized (hPAM4; clivatuzumab) versions provided by Immunomedics, Inc. (Morris Plains, NJ). All other MAbs were murine IgG. Mouse ascites fluids containing MAbs 21 M1, 45 M1, 62 M1 and 463 M1 were kindly provided by Dr. J. Bara, INSERM, Paris, France. PAM4 antibodies and ascites fluid containing an anti-alpha-fetoprotein antibody, employed as a negative control for the blocking studies (reactive with Hep-G2, hepatocellular carcinoma cells) were provided by Immunomedics, Inc. (Morris Plains, NJ). A rabbit polyclonal anti-CPM1 [14,16] IgG served as the positive control with detection by a horseradish peroxidase (HRP)-labeled donkey anti-rabbit IgG (Jackson ImmunoResearch, West Grove, PA).

**Table 1:** Monoclonal antibodies used in the current studies

**Antigen**	**Clone name**	**Source**
MUC1	MA5	Immunomedics
MUC1	KC4	Immunomedics
MUC1	CM1	Gene Tex
MUC2	994/152	Abcam
MUC3	M3.1	Abcam
MUC3	M3A	LifeSpan Bio
MUC4	8G7	Santa Cruz Biotech
MUC5AC	2-11 M1	Santa Cruz Biotech
MUC5AC	45 M1	Santa Cruz Biotech
MUC5AC	CLH2	Santa Cruz Biotech
MUC16	X306	Novus Bio
MUC16	X325	Abcam
CEACAM5	MN14	Immunomedics
CEACAM6	MN15	Immunomedics
CA 19-9	CA 19-9	Santa Cruz Biotech

Immunomedics, Inc. – Morris Plains, NJ; GeneTex – Irvine, CA; Abcam – Cambridge, MA; LifeSpan Biosciences, Inc. - Seattle, WA; Santa Cruz Biotechnology, Inc. - Santa Cruz, CA; Novus Biologicals – Littleton, CO.

### Enzyme immunoassay

Procedures have been described for both indirect and sandwich enzyme immunoassays [14,16]. For indirect immunoassays, primary MAbs were used at a concentration of 10 μg/mL to provide high sensitivity for signal detection. For sandwich immunoassays, the capture MAb was coated onto the wells at a concentration of 10 μg/mL, followed by the addition of the CPM1 antigen at various concentrations up to 10 μg/mL. The MAb probe was then added at a high concentration of 10 μg/mL for detection of response to captured antigen. Secondary HRP-labeled anti-species-specific IgG (Jackson ImmunoResearch, West Grove, PA) was evaluated initially to determine optimum concentrations for use in the assay (usually 1:1000 or 1:2000). MAb inhibition studies were performed by adding the inhibiting MAb to wells coated with CPM1 antigen, starting at a high concentration of 100 μg/mL of pure MAb or 1:10 dilution of ascites fluid, and titrating to lower amounts. After incubating with the inhibiting antibody at 37°C for 1 h, the plates were washed, and hPAM4 added to the wells at a concentration of 0.25 μg/mL. hPAM4 binding was then detected with a secondary probe, HRP-labeled anti-human IgG conjugate.

### Recombinant expression of MUC5AC C-terminal domains

The plasmid of pSM-MUC5AC-CH-long, encoding a signal sequence, a Myc tag, the complete human MUC5AC C-terminal cysteine-rich part, and a His tag, is a gift from Dr. Gunnar C. Hansson (University of Gothenburg, Gothenburg, Sweden) [28]. CFPAC-1 cell line was obtained from American Type Culture Collection (Manassas, VA) and maintained in ATCC-formulated Iscove's Modified Dulbecco's Medium plus 10% FBS at 37°C in 5% CO_2_. Transfection was performed using Lipofectamine 2000 (Life Technologies, Grand Island, NY) when cells reached about 85% confluent. Seventy-two hours later, the spent medium was collected and 10-fold concentrated using 10 kD Amicon ultrafiltration membrane (EMD Millipore, Billerica, MA). The recombinant proteins were purified using an anti-Myc column (Vector laboratories, Burlingame, CA) from the concentrated medium.

### SDS-PAGE and western-blot

SDS-PAGE was performed under non-reducing conditions using 4-20% Tris-Glycine gels (Lonza, Allendale, NJ) at 125 V for about 2 h. Resolved proteins were transferred onto a nitrocellulose membrane using the Mini Trans-Blot® cell system (Bio-Rad Laboratories, Hercules, CA) at 100 V for 1 h. To examine the identity of recombinant proteins, triplicate samples were run in the same gel and membrane with transferred samples cut into three pieces for probing with HRP-anti-Myc, HRP-hPAM4, and 45 M1 plus HRP-GAM, respectively. The signals were developed with SuperSignal™ West Dura Chemiluminescent Substrate (Thermo Fisher Scientific, Waltham, MA).

## Abbreviations

CPM1: Capan-1-mucin fraction 1; HRP: Horseradish peroxidase; EIA: Enzyme immunoassay; hPAM4: Humanized PAM4 IgG; IPMN: Intraductal papillary mucinous neoplasia; MAb: Monoclonal antibody; mPAM4: Murine PAM4 IgG; PanIN: Pancreatic intraepithelial neoplasia; PDAC: Pancreatic ductal adenocarcinoma.

## Competing interests

David M. Goldenberg and Donglin Liu have a financial interest in Immunomedics, Inc., which owns rights to the PAM4 antibodies. David M. Goldenberg and David V. Gold are patent inventors. Guy Newsome declares no competing interests.

## Authors’ contributions

DVG designed the study, DVG, GN, and DL performed the experiments and analyzed the data. DVG and DMG interpreted the results and wrote the paper. All authors read and approved the final manuscript.
